# Facilitating author-driven, machine-readable descriptions with the new minISA metadata format

**DOI:** 10.1038/s41597-020-00641-9

**Published:** 2020-09-15

## Abstract

Today, the journal is announcing a series of improvements to the way we collect and expose the machine-readable metadata supporting our publications. These innovations are designed to make it easier for our authors to engage in the metadata creation process, and easier for users to access and benefit from the information we capture.

Since launch, *Scientific Data* has provided a machine-readable metadata file alongside each published Data Descriptor to summarize key characteristics about the data being described in the article^1^. Until August 2019, the metadata files provided for every Data Descriptor were based on the full ISA-Tab format^2,3^ and were searchable via the ISA-Explorer discovery tool^4^. Although the ISA-Tab format was initially developed with biological assays in mind, it is a highly flexible format that allowed the journal to represent the generation of data in a machine-readable manner across a number of research disciplines.

The data sharing and management landscape has changed dramatically since the journal first launched. In the last six years, we have seen the emergence of the FAIR data concept^5^, the maturation of the data repository ecosystem, the emergence of repository indexes (e.g. https://fairsharing.org/ & https://www.re3data.org/), data indexing services (e.g. https://datamed.org/ & https://datasetsearch.research.google.com/), and advances in formal data citation infrastructure^6^. With machine-readable metadata now available from a wider range of sources, we have sought to focus our efforts on providing lighter metadata files that bridge the gap between citation-level metadata and the richer highly-structured information provided by data repositories.

To this end, we have worked with the ISA coordinator group (https://isa-tools.org) to update our Data Descriptor machine-readable metadata format. The changes are twofold. We upgraded the representation from tabular to JSON-LD format (https://json-ld.org/) and reduced the set of elements in the metadata. Importantly, we have kept the cross-references to the relevant data records in repositories, where users can access the full repository-level metadata and the data files.

To develop this new lighter-weight metadata format we used the existing ISA-JSON format as a starting point, and extracted the metadata elements that focus on describing: how and where data were measured, what experimental factors were varied in the study, and related publications that demonstrate usage of the data. We then anchored these elements to Schema.org types to make them compatible with other semantic web metadata sources (https://schema.org/). Metadata in the new minISA format^7^ are now available for all Data Descriptors published since September 2019. The metadata files are hosted in figshare, receive their own distinct digital object identifiers, and are accessible directly from each Data Descriptor or via figshare’s API.

The minISA format has been implemented in a new custom built webtool developed in partnership with figshare. Authors will be sent a link to the tool at an appropriate point during the review process.

From today, all Data Descriptor authors will be asked to submit information about their data via the tool during the editorial and review process. Our in-house curation team will continue to work with authors of accepted manuscripts to finalize the metadata, ensuring the use of controlled vocabulary and ontology terms wherever possible. The curation team will also continue to carry out their usual checks to ensure the identification and remedy of any errors that may have been overlooked during peer review, and to improve dataset reusability^8^. While minISA will be used for all of our machine readable metadata going forward, our past ISA-Tab formatted metadata files remain available from each online Data Descriptor. We have also permanently archived a copy of the full set of 852 *Scientific Data* ISA-Tab files at figshare^9^.

Metadata can be used to enhance the discovery and reuse value of the data described, thereby improving the FAIRness of the data. The metadata files associated with each Data Descriptor article have always been available for use by all under the CC0 waiver^1^ and can be downloaded directly from each Data Descriptor article online. Data discovery infrastructure is still in its infancy, and we aim to continue to play an active role in this space. We are excited to see what the future holds for *Scientific Data*’s minISA metadata files and look forward to the next innovations in data publishing.
